# Early vestibular rehabilitation training of peripheral acute vestibular syndrome—a systematic review and meta-analysis

**DOI:** 10.3389/fneur.2024.1396891

**Published:** 2024-05-30

**Authors:** Helle Elisabeth Agger-Nielsen, Thomas Stig Grøndberg, Gabriele Berg-Beckhoff, Therese Ovesen

**Affiliations:** ^1^Department of Otorhinolaryngology, Hospital of Southwest Jutland Esbjerg, Esbjerg, Denmark; ^2^Department of Regional Health Research, University of Southern Denmark, Odense, Denmark; ^3^Department of Physiotherapy Education, University College South Denmark, Esbjerg, Denmark; ^4^Health Promotion, University of Southern Denmark, Esbjerg, Denmark; ^5^Department of Otorhinolaryngology, Regional Hospital Gødstrup, Herning, Denmark

**Keywords:** acute vestibular syndrome, acute dizziness, vestibular rehabilitation, acute unilateral vestibular loss, vestibular neuritis, systematic review, meta-analysis, corticosteroid

## Abstract

**Objective:**

This study aimed to investigate the impact of early vestibular rehabilitation training combined with corticosteroids initiated within 2 weeks, compared with corticosteroid treatment, after the peripheral acute vestibular syndrome (pAVS) onset.

**Data sources:**

PubMed, CINAHL, EMBASE, and SCOPUS. From inception to January 24, 2024. The International Prospective Register of Systematic Reviews approved this study (CRD42023422308).

**Results:**

Five studies involving 235 patients were included in this systematic review and meta-analysis. The subjective outcome measure Dizziness Handicap Inventory (DHI) was pooled for a meta-analysis and was statistically significantly in favor of early vestibular rehabilitation training (early VRT) plus corticosteroids compared with corticosteroids alone: at one-month follow-up (*p* = 0.00) and 12 months follow-up (*p* = 0.01). DHI was a critical outcome for measuring the differences in effect of early VRT. The objective outcome measures of caloric lateralization, cervical vestibular-evoked myogenic potentials, and posturography were gathered for a narrative synthesis.

**Conclusion:**

This meta-analysis showed that early VRT in combination with corticosteroids was more effective for treating pAVS than corticosteroid treatment alone. No adverse effects were reported for early VRT.

## 1 Introduction

Acute vestibular syndrome (AVS) is defined as the acute onset of continuous vertigo lasting >24 h. AVS is often associated with nausea, vomiting, and head motion intolerance (1). In the United States, acute vertigo is estimated to account for 4% of patients admitted to emergency departments (2). Furthermore, vertigo is extremely distressing for patients and may lead to hospitalization.

There are two types of AVS: central AVS (cAVS), which is caused by stroke, and peripheral AVS (pAVS), which is caused by various influences of the vestibular organ in the inner ear and/or vestibular nerve. The pAVS includes several specific diagnoses, including vestibular neuritis, Meniere’s disease, perilymphatic fistulas, and labyrinthitis. The specific cause of AVS may not be evident during an acute diagnostic workup; therefore, the terms p-AVS and c-AVS were introduced to differentiate the two conditions. However, this distinction is extremely important because of the potentially fatal outcome of overlooking stroke (3–6). Accordingly, subdivision into two entities also determines the treatment.

Regarding cAVS, some evidence supports the effectiveness of early rehabilitation (7). In pAVS, early rehabilitation is only sporadically described in available research and guidelines (8, 9). Hypothetically, vestibular rehabilitation, that is, physical exercise developed to stimulate the vestibular system, may have a significant effect on the outcome of pAVS. A Cochrane review in 2015 on unilateral peripheral vestibular dysfunction found that vestibular rehabilitation was more effective than control or sham interventions in improving objective and subjective reports of symptomatology. However, only a few of these studies have focused on acute vestibular dysfunction and mainly covered cases of surgery for vestibular neuromas and BPPV (8). Additionally, the focus was on vestibular rehabilitation in general rather than early-vestibular rehabilitation training (early VRT). To our knowledge, no other systematic review on early VRT for pAVS exists. Other related reviews concern vestibular rehabilitation/vestibular rehabilitation therapy for vestibular neuritis/vestibular neuronitis (9–11). The lack of knowledge regarding early VRT for pAVS, other than iatrogenic dysfunction and BPPV, raises the following question:

What are the effects of early VRT combined with corticosteroid, initiated within 2 weeks, on recovery from pAVS?

This systematic review aimed to investigate the outcomes of vestibular rehabilitation combined with corticosteroids initiated within 2 weeks of pAVS onset compared with corticosteroids.

## 2 Methods

The Preferred Reporting Items for Systematic Reviews and Meta-Analyses (PRISMA) guidelines were used for this systematic review (12). This study was registered with PROSPERO (CRD42023422308). Approval of the study by the institutional review board or the Danish Research Ethics Committee was not required.

### 2.1 Study inclusion

Randomized clinical trials (RCTs) involving patients treated with early VRT for pAVS were included (Table 1). Early VRT can be delivered in combination with corticosteroids, as this is regarded standard care. The Covidence data-screening tool (13) was used to screen the references.

**Table 1:** Study inclusion criteria according to PICO: population, intervention, comparators, and outcome.

Population	Adult (≥ 18 years) patients undergoing vestibular rehabilitation owing to peripheral acute vestibular syndrome (pAVS). In some adolescents, the development of the vestibular function is not completed. Thus, the cut-off is set at 18 years of age.
Intervention	Any intervention with early vestibular rehabilitation training after pAVS in combination with any type of corticosteroids. Vestibular rehabilitation includes any training program developed by physiotherapists to stimulate the vestibular system, initiated within 2 weeks after onset of pAVS.
Comparators	Patients undergoing no intervention, receiving standard care, and/or systemic medical treatment.
Outcome	Any outcome measuring effects of vestibular rehabilitation: Vestibular (objective tests): Post-urography (measures overall balance); the Video Head Impulse Test (v-HIT, measures the vestibulo-ocular reflex); video nystagmography (VNG) (measures eye movements); caloric response/caloric irrigation (measures preponderance and unilateral weakness in vestibulo-ocular response); ocular vestibular evoked myogenic potentials (oVEMP) and cervical vestibular evoked myogenic potentials (cVEMP) (measure unilateral vestibular loss). Subjective patient-reported outcome measures (PROMs): the Dizziness Handicap Inventory (DHI); the Hospital Anxiety and Depression Scale (HADS); the California Los Angeles Dizziness Questionnaire (CLADQ); Visual Analog Scale (VAS); Visual Analog Scale for Anxiety (VASA); the European Evaluation of Vertigo Scale (EEVS), or other symptom burden scales.

### 2.2 Study exclusion

Studies of cAVS, BPPV, or chronic dizziness were excluded. Despite its acute onset, BPPV is considered an episodic syndrome and was excluded from the review because repositioning maneuvers are the treatment of choice. RCTs with less than 30 participants, representing a minimum of 15 participants in each group, were excluded and so was non-RCTs.

### 2.3 Search strategy and eligibility of studies

On March 7, 2023, we conducted an initial literature search of the PubMed, EMBASE, CINAHL, and SCOPUS electronic bibliographic databases. The search strategy included both MeSH terms and free text searches for acute dizziness, acute vertigo, dizziness, vertigo AND vestibular rehabilitation, therapy, physical therapy, AND randomized controlled trial OR RCT. Further search restrictions included English language and publication years after 1999. The search protocols are presented in Supplementary Table S1.

We searched for relevant RCTs assessing the effect of early VRT in combination and compared with corticosteroids, placebo, or no intervention in patients with pAVS. The last search date was January 24, 2024, and no studies were added to the interval separating the two searches.

### 2.4 Data extraction and management

Data were extracted from five eligible publications by HAN and TG, and when available, mean differences and standard deviations (SD), 95% confidence intervals, and *p* values were extracted. The data were pooled for meta-analysis of the Dizziness Handicap Inventory (DHI). For the remaining outcomes, the data could not be numerically synthesized owing to considerable heterogeneity, between-study variations in outcome measures and follow-up times, and differences in the rehabilitation protocols.

The Consensus on Exercise Reporting Template (CERT) (14) was applied to evaluate whether the applied vestibular rehabilitation protocols were completely and explicitly reported (Supplementary Table S2).

The risk of bias was evaluated by the Cochrane Collaboration’s Tool for assessing the Risk Of Bias (ROB-2) (15).

To assess study quality and evidence, we adopted the Grading of Recommendations Assessment, Development, and Evaluation (GRADE) (16) approach. This approach ensures a transparent and structured process for developing and presenting a summary of the evidence.

Consensus on the GRADE quality of evidence and importance of outcomes in the included RCTs was obtained by HAN and TG, and TO made the final decision in the case of discrepancies.

### 2.5 Statistical analysis

A random-effects model was used for the meta-analysis. Meta-analysis was done on DHI using means and standard deviations, version 18.0 STATA. A random-effects model was chosen to ensure more trustworthy estimates despite large heterogeneity at baseline measurement of DHI. A random effect model takes into account that the standard error of the estimate differs between the studies. For DHI, an analysis on heterogeneity was conducted. A narrative synthesis of the following outcomes was performed for the remaining data: caloric lateralization, vestibular-evoked myogenic potentials (VEMP), posturography, visual analog scale (VAS), and Dynamic Gait Index (DGI).

## 3 Results

### 3.1 Study selection

**Figure 1:**
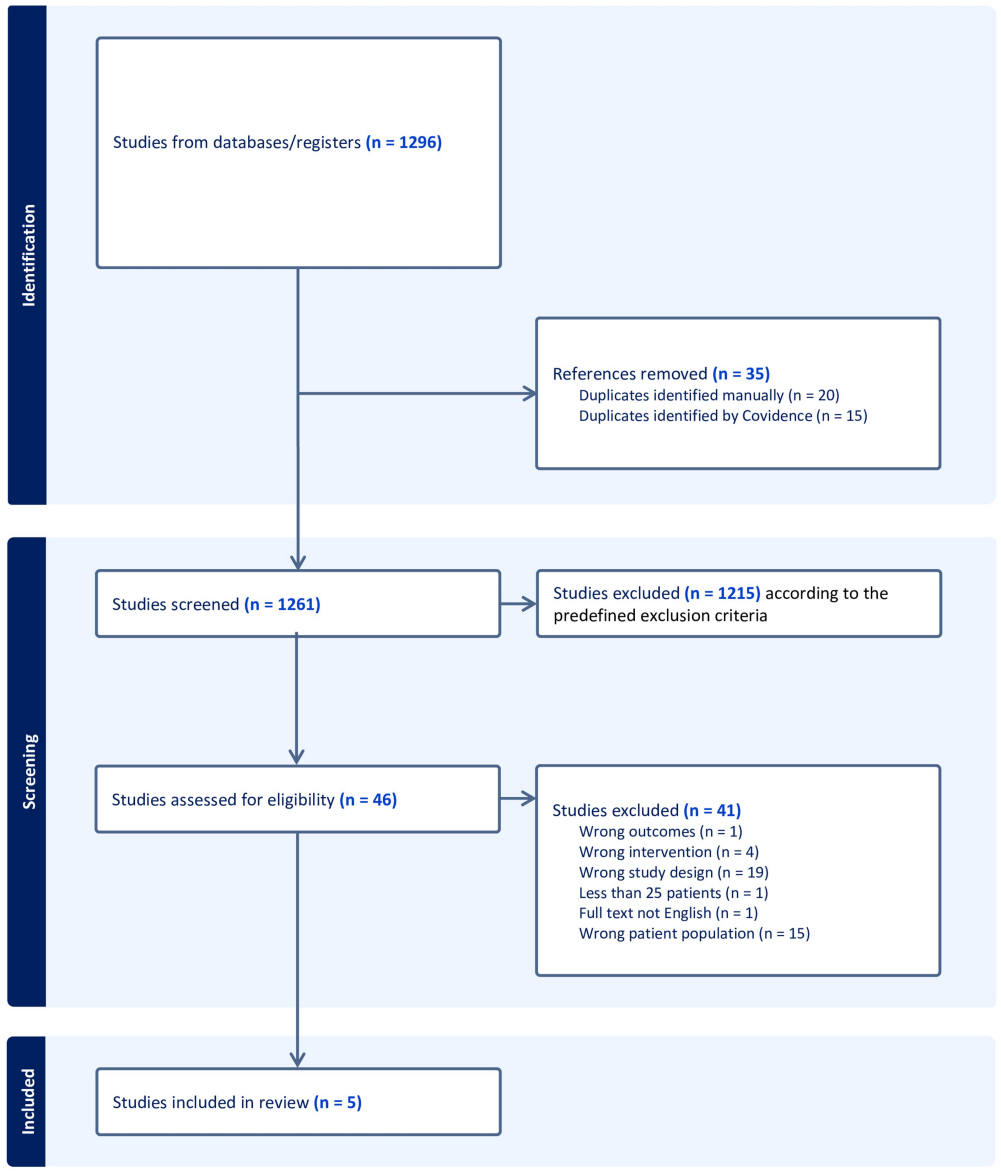
PRISMA flowchart.

The preliminary search identified 1,296 publications, of which 35 were excluded, as described in the PRISMA (12) flowchart in Figure 1. Thus, 1,261 publications were screened by title and abstract by HAN and TG using the Covidence software. We excluded 1,215 publications based on the predefined exclusion criteria. Subsequently, 46 publications were read in full by HAN and TG. Thereafter, the reference lists of the publications were screened for relevant studies. In cases of disagreement regarding eligibility, TO made the final decision on whether to include or exclude the study. A total of 41 publications were excluded from the analysis. Thus, five publications conducted by HAN, TG, and TO were included in the final review.

### 3.2 Study characteristics

Table 2 provides an overview of the five included RCTs (17–21) of early VRT plus corticosteroids initiated within 2 weeks after pAVS onset, with a focus on the intervention arms, outcome measures, and results reported. In Supplementary Table S2 the baseline characteristics of the included studies are described. There are no differences between the groups at baseline, in any of the studies included.

**Table 2:** Overview of the included randomized controlled trials (RCTs) of the early VRT in case of peripheral acute vestibular syndrome (pAVS).

Author	No	Medical treatment	Baseline assessment days from onset	CERT score	Intervention arms	Outcomes	Results
Goudakos 2014	40	Dexamethasone (IV)^a^	4	9	1. eVRT + late steroid 2. Steroid	DHI, EEV, Caloric (lateralization >25%), abnormal VEMP	No SS difference at 1-, 6- and 12-month follow-up
Ismail 2018	60	Methylprednisolone (OR)^b^	4	3	1. eVRT 2. Steroid 3. eVRT + steroid	DHI, caloric (lateralization >20%), cVEMP asymmetry	No SS difference at 1-, 3-, 6-, and 12-month follow-up
Marioni 2013	30	Betamethasone disodium phosphate (IV)^c^	14	14	1. eVRT + steroid 2. Steroid 3. Healthy volunteers	Caloric (lateralization >50%), post-urography: limits of stability (LOS), mCTSIB	eVRT versus steroid was SS after 6 weeks in six post-urographic parameters (*p* = 0.03; 0.00000009; 0.04; 0.02; 0.05; 0.03)
Teggi 2009	40	Prednisolone (OR)^d^	4	7	1. eVRT + steroid 2. Steroid	DHI, DGI, VAS—Anxiety, post-urography	SS in favor of eVRT at: 1-month follow-up: ∆-percentage DHI total (*p* = 0.002), ∆-percentage VAS-A (*p* = 0.001)
Tokle 2020	65	Prednisolone (OR)^e^	Within 7	17	1. eVRT + steroid 2. Steroid	Perceived dizziness, walking speed, standing balance, DHI, VAS-A*, VAS-B, VAS-C, HADS, VSS, UCLA-DQ	SS in favor of the eVRT at: 3-month follow-up: perceived dizziness (*p* = 0.007) 12-month follow-up: Perceived dizziness (*p* = 0.001), HADS (*p* = 0.039), DHI (*p* = 0.049), and VAS-C (*p* = 0.012).

SS, Statistically significant; NS, Not significant; *p*, *p*-value below 0.05 (is considered statistically significant); eVRT, Early vestibular rehabilitation training; Steroid, Treatment with corticosteroids; OR, Orally; IV, Intravenously; DHI, Dizziness Handicap Inventory; EEV, European Evaluation of Vertigo Scale; DGI, Dynamic Gait Index; c-VEMP, cervical Vestibular Myogenic Potentials; VEMP, Vestibular Myogenic Potentials; VAS-A, Visual Analog Scale – Anxiety (VAS-A*, VAS-B, VAS-C under different positions/movements in Tokle); mCTSIB, modified Clinical Test of Sensory Measures; HADS, Hospital Anxiety and Depression Scale; VSS, Vertigo Symptoms Scale; UCLA-DQ, University of California Los Angeles Dizziness Questionnaire.

a Dexamethasone sodium phosphate 24 mg/day (IV), tapered over 7 days. On discharge from the hospital, all patients received 14 days of dexamethasone sodium phosphate 2 mg (OR) per day tapered down (late steroid).

b Methylprednisolone 20 mg (OR) three times daily for 1 week, tapered over the second week.

c Betamethasone disodium phosphate 4 mg (IV) a day for 7 days.

d All patients received prednisolone 50 mg for 2 days, 25 mg for 3 days, and 12.5 mg for 2 days (OR).

e Prednisolone 60 mg for 5 days; and 50, 40, 30, 20, and 10 mg each for 1 day.

The specific protocols for vestibular rehabilitation are described in Supplementary Table S3. In Table 2, the total CERT scores are displayed; the results range from 3 to 17. The maximum possible score was 19 points. In the included studies, one intervention arm received corticosteroid treatment, and one arm received early VRT plus corticosteroid treatment. Only one study by Ismail et al. included an intervention arm with early VRT as a stand-alone treatment, and one study by Marioni et al. included an intervention arm with healthy volunteers.

No studies were found that compared early VRT without corticosteroids with no treatment. A study from Yoo et al. compared corticosteroid therapy with no medical treatment, with both groups being instructed to do vestibular exercises plus taking *Ginkgo biloba*, and found that there was no additional effect of corticosteroids (22). Another study by Kammerlind et al. found no differences in outcome for groups performing home training and one group performing additional physical therapy (23). Yet, another study by Strupp et al. found that corticosteroid was superior to valacyclovir in treating vestibular neuritis (24).

### 3.3 Risk of bias

The risk of bias was evaluated using ROB-2, and the domains of the included RCTs were assessed (Table 3). Blinding of patients and personnel was difficult, and four of five studies fail. One in four fail in blinding statistician. Two out of five studies fail in describing if the statistician was blinded. One study is at risk of selective outcome reporting due to missing description of dropouts.

**Table 3:** Risk of bias of the included studies investigating the early vestibular rehabilitation training (eVRT) in peripheral acute vestibular syndrome (pAVS).

Author	Sequence generation	Allocation concealment	Blinding of participants and personnel for all outcomes	Blinding of outcome assessors for all outcomes	Incomplete outcome data for all outcomes	Selective outcome reporting	Other sources of bias
Goudakos 2014	Block randomization	Computer-generated block randomization	Single blinding	The investigator who performed follow-up evaluations of outcomes was masked to the treatment groups	40/40 completed follow-up	52 patients assessed for eligibility, 6 not meeting the inclusion criteria, 6 refused to participate, and 40 randomized.	Low
Ismail 2018	Randomly assigned	Randomly assigned	No blinding	The investigator who performed the follow-up evaluations of outcomes was masked to the patient’s allocation to the treatment groups	*No description of 24/84 patients dropping out at 6 and 12 months. “…some of the patients refused follow-up and others informed us by phone that they became well…”*	No description of the dropouts and selective outcome reporting is a risk.	Low
Marioni 2013	Patients were randomized in a 1:1 ratio	The randomization schedule was computer-generated	No blinding	Outcome assessors—Blinding was not described	All the patients who were enrolled completed the study	All outcomes are accounted for	Low
Teggi 2009	The randomization was performed by a computer-generated sequence	The randomization was performed by a computer-generated sequence	No blinding	No blinding for outcome assessors	No dropouts	All outcomes are accounted for	Low
Tokle 2020	Randomly allocated 1:1.	Computer random number generator	Assessments and intervention administration were done unblinded	The statistician conducting the statistical analyses was blinded to group allocation	65 patients were randomized, 56 patients gave consent, 52 completed follow-up, and all dropouts were accounted for	The trial and the principal analyses were based on the “intention to treat” principle	Low

### 3.4 Results of the syntheses

**Figure 2:**
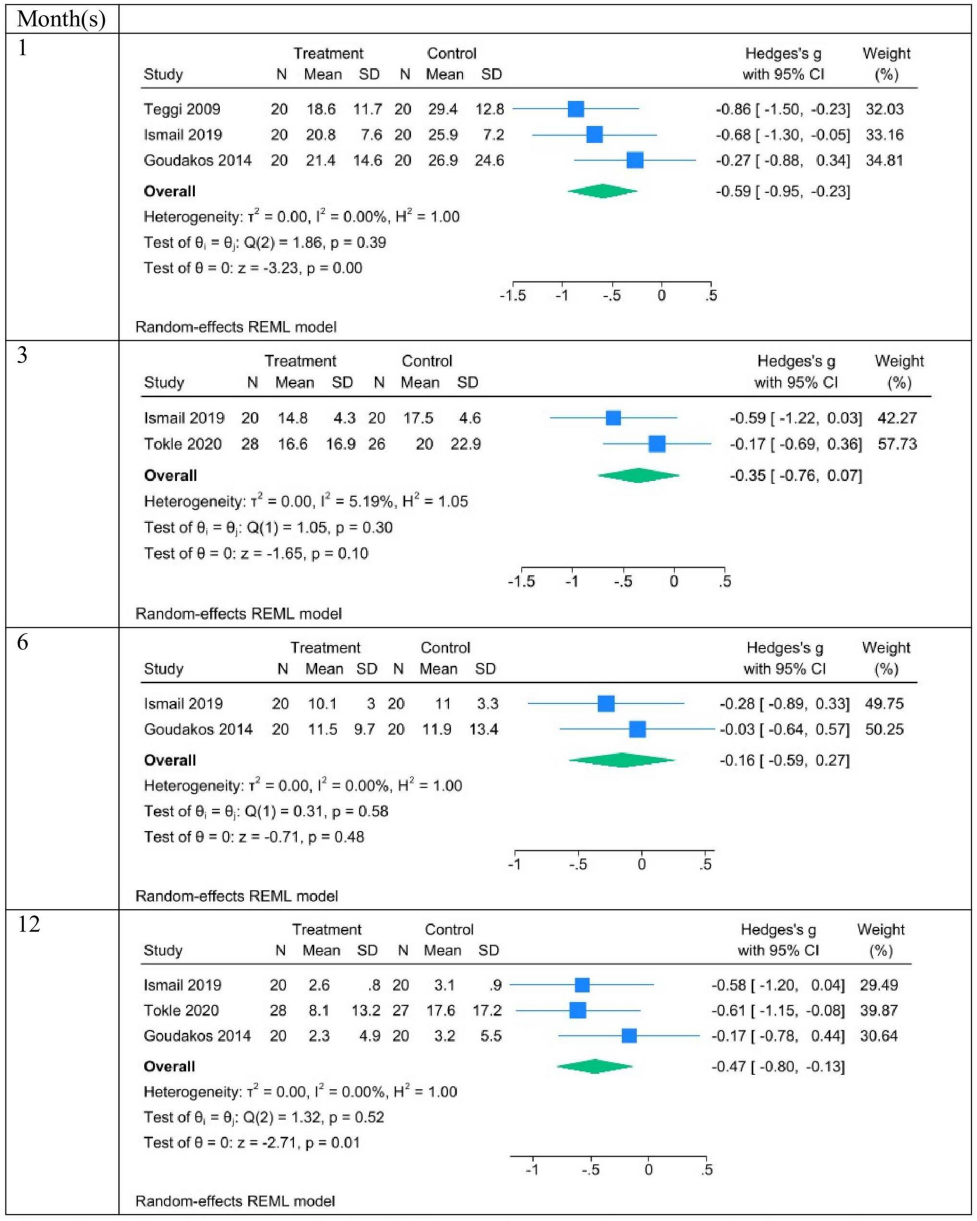
Forest plots of the difference between the treatment (early VRT plus corticosteroid) and control (corticosteroid) groups for all follow-up months.

As DHI was included as an outcome measure in four studies, a meta-analysis of the extracted DHI results was performed (Table 4; Figure 2). Table 4 presents the meta-analyses of mean DHI in different time points. In all follow-up periods the means of DHI are very similar, demonstrating homogeneity. However, in the baseline, high heterogeneous results of DHI are given. Heterogeneity was high at baseline 98% in early VRT plus corticosteroid group and corticosteroid group; however, very low at any of the follow-ups.

**Table 4:** Descriptive results from the dizziness handicap inventory (DHI) in different studies.

Study	Baseline	1 month	3 months	6 months	12 months
Early VRT plus corticosteroidgroup					
Teggi 2009	51.2 (8.9)	18.6 (11.7)	–	–	–
Ismail 2019	96.3 (2.5)	20.8 (7.6)	14.8 (4.3)	10.1 (3.0)	2.56 (0.8)
Tokle 2020	–	–	16.6 (16.9)	–	8.1 (13.2)
Goudakos 2014	95.30 (2.6)	21.43 (14.6)	-	11.50 (9.7)	2.25 (4.9)
Meta-analysis	82.1 (1.1)	20.4 (0.5)	14.9 (0.3)	10.3 (0.2)	2.6 (0.1)
*I*^2^ [in %]	98.03	0.00	0.00	0.00	0.00
Corticosteroid group					
Teggi 2009	50.7 (8.7)	29.4 (12.8)	–	–	–
Ismail 2019	95.9 (3.1)	25.9 (7.2)	17.5 (4.6)	11.0 (3.3)	3.1 (0.9)
Tokle 2020	–	–	20.0 (22.8)	–	17.6 (17.2)
Goudakos 2014	96 (3.7)	26.94 (24.6)	–	11.88 (13.4)	3.17 (5.5)
Meta-analysis	82.0 (1.1)	26.7 (0.5)	17.6 (0.3)	11.1 (0.2)	3.1 (0.1)
*I*^2^ [in %]	98.10	0.00	0.00	0.00	0.00

The table presents the mean (standard deviation) and summarized results via meta-analysis (random effect models) and their estimated heterogeneity (*I*^2^).

Figure 2 shows the meta-analyses of DHI differences between the treatment (early VRT plus corticosteroid) and control (corticosteroid) groups at all follow-ups. The estimated effect was a statistically significant reduction of DHI in the early VRT plus corticosteroid group after 1 month −0.59 CI(−0.95, −0.23); *I*^2^ = 0.0%; *p* = 0.00. Moreover, the reduction was also statistically significant after 12 months of follow-up. The estimated effect was −0.47 CI(−0.8, −0.13); *I*^2^ = 0.0%; *p* = 0.01. In the follow-up after three and 6 months, with only two studies included, a reduction was found, but it was not significant. That is, in pAVS, early VRT plus corticosteroids was more effective than corticosteroids, as measured after 1 and 12 months, in improving subjective dizziness measured with DHI.

### 3.5 Results of GRADE

Table 5 summarizes the relevant outcomes investigated in these studies. The DHI was the only questionnaire used in more than one study (see the DHI meta-analysis) (Figure 2).

**Table 5:** Summary of findings: GRADE on patient-important outcomes.

**Author:** Helle Elisabeth Agger-Nielsen. **Question:** Early VRT (eVRT) plus corticosteroid compared to corticosteroid (steroid) for peripheral Acute Vestibular syndrome (pAVS). **Setting:** Hospital.									
Certainty assessment							Impact	Certainty	Importance
No. of studies	Study design	Risk of bias	Inconsistency	Indirectness	Imprecision	Other considerations			
Subjective dizziness, DHI									
4	Randomized trials	Not serious	Not serious	Not serious	Serious^a^	None	1 month: The estimated effect was a significant reduction in DHI in eVRT+steroid versus steroid, −0.59 CI (−0.95, −0.23), P = 0.00 12 month: The estimated effect was a significant reduction in DHI in eVRT+steroid versus steroid, −0.47 CI (−0.80, −0.13), P = 0.01	⨁⨁⨁◯ Moderate	Critical 8
Objective dizziness, caloric lateralization									
2	Randomized trials	Not serious	Not serious	Not serious	Serious^b^	None	1**-**month ∆-percentage: G: eVRT+steroid 32.4% vs. steroid 33.6% I: eVRT+steroid 41.3% vs. steroid 38.1%, 6**-**month ∆-percentage: G: eVRT+steroid 55% vs. steroid 60%, I: eVRT+steroid 65.2% vs. steroid 65.4%, 12**-**months ∆-percentage: G: eVRT+steroid 75.5% vs. steroid 69.7%, I: eVRT+steroid 79.6% vs. steroid 73.1%.^c^	⨁⨁⨁◯ Moderate	Critical 8
Objective dizziness, cVEMP									
2	Randomized trials	Not serious	Not serious	Serious^d^	Serious^e^	None	Abnormal at baseline: eVRT plus steroid 15/40 vs. Steroid 12/40. Abnormal at 1 month: eVRT plus steroid 11/40 vs. steroid 10/40. Abnormal at 12 months: eVRT plus steroid 0/40 vs. steroid 0/40	⨁⨁◯◯ Low	Not important 3
Objective dizziness, posturography									
2	Randomized trials	Not serious	Serious^f^	Not serious	Serious^g^	None	T: Quotients Q1 (eyes open/eyes closed) (*p* = 0.01), Q2 (eyes open on foam/eyes closed on foam) (*p* = 0.01), and Q3 (eyes open/eyes closed on foam) (*p* = 0.01). M: The Modified Clinical Test of Sensory Organization and Balance (mCTSIB), at 6 weeks follow-up was significant in the following positions: open eyes/foam surface (OEFS) (*p* = 0.03) and closed eyes/foam surface (CEFS) (*p* = 0.00000009).^h^^,^^i^	⨁⨁◯◯ Low	Critical 7

CI, confidence interval; eVRT, Early vestibular rehabilitation training; Steroid, corticosteroid.

a Large heterogeneity at baseline.

b Large differences in cutoff for normal versus abnormal test in caloric lateralization.

c Goudakos et al.

d Not all the patients included had an abnormal test at baseline.

e Few abnormal tests, render this outcome less reliable in detecting an improvement in objective dizziness.

f 2 different platforms measuring different modalities: dynamic posturography versus static posturography.

g Many different parameters are measured, but the connection to subjective dizziness via one strong parameter has yet to be found.

h Teggi et al.

i Marioni et al.

The subjective outcome measures were addressed for the GRADE evaluation:

DHI was downgraded due to large heterogeneity at baseline. The certainty of evidence was moderate, and the importance of the outcome was critical.

The other outcome measures investigated in these studies have not been described in further detail. Subjective dizziness outcome measures, including the Hospital Anxiety and Depression Scale, Los Angeles Dizziness Questionnaire, VAS, VAS for Anxiety, and European Evaluation of Vertigo Scale questionnaires, were applied only in one study, and data pooling was therefore impossible. The Perceived Dizziness Test was used in only one study; therefore, pooling was irrelevant.

The objective outcome measures were addressed for the GRADE evaluation:

Caloric lateralization was downgraded due to imprecision in different cutoff values. The certainty of evidence was moderate, and the importance of the evidence was critical. In caloric lateralization, the data from two studies are presented as ∆-percentages from baseline to 1, 6, and 12 months. The ∆-percentage may seem significant at 12 months (75.5% vs. 69.7%); however, the different cut-off values and limited data from only these two studies did enable further analysis. Goudakos et al. considered caloric lateralization abnormal if it was >25%, and Ismail et al. considered a 20% lateralization or higher abnormality. Marioni et al. reported that more than 50% of the cases of vestibular weakness were abnormal. Owing to these large differences in cut-off values, a meta-analysis could not be conducted.

Cervical vestibular evoked myogenic potential (cVEMP) was downgraded due to indirectness and imprecision. The large number of normal tests in the affected patients renders this outcome useless for detecting of change in dizziness. The certainty of the evidence was low, and the outcome was not important. The cVEMP data of 80 patients with pAVS are reported in Supplementary Table S3.

Posturography was downgraded due to different ways of measuring, with two different platforms. This measure was further downgraded due to imprecisions as multiple parameters instead of one solid parameter are at play. The certainty of the evidence is low, and the importance of the evidence was critical. The posturographic outcome measures were significant and in favor of the early VRT group for several parameters in both studies. However, between-study differences in the outcomes obtained from posturography rendered further analysis impossible.

DGI was used in only one study; therefore, data pooling was not possible.

## 4 Discussion

According to the meta-analysis of differences, early VRT plus corticosteroids reduced DHI compared with corticosteroid in pAVS. This effect was significant at 1 and 12 months. Due to the small number of studies (reduced power), the meta-analyses revealed no significant reduction in the follow-up of at 3 and 6 months. The essence of the present systematic review was that the GRADE evaluation suggested that early VRT and corticosteroids affect objective outcome measures. The GRADE evaluation process underlines the importance of remaining attentive to minor potential effects and considering all results that could contribute to the evaluation. However, none of these effects were significant except for DHI.

The number of included studies was consistent with that of other studies on related topics. Hidayati et al. (10) included four RCTs in a systematic review on vestibular rehabilitation and corticosteroids for vestibular neuritis, and García-Mûnoz et al. included five RCTs in a systematic review on vestibular training for patients with multiple sclerosis (25). In a systematic review by Chen et al. (9) on vestibular rehabilitation training combined with anti-vertigo drugs for vertigo and balance function in patients with vestibular neuritis, 18 of the 21 studies originated from China and could be found only via searches of Chinese literature databases. Some relevant studies may have been missed in the present review because they were Chinese.

The five RCTs reported homogeneous intervention groups. Geographically, the five studies were conducted in various countries, from Norway to Egypt, which might have posed a risk of cultural bias. Thus, the review by Chen et al. mentioned above had results that were in line with ours in terms of DHI meta-analyses.

Generally, the risk of bias was low in the included studies. The lack of blinding of the intervention was considered a bias. However, blinding of physical exercise is almost impossible. None of the included studies reported how and by whom the patients were screened for inclusion. The studies only included patients who had spontaneous nystagmus for several days, with a baseline assessment on day 4–14. Thus, milder and less grave cases of pAVS were missed because of the disappearance of nystagmus. Remission of spontaneous nystagmus associated with vestibular neuritis often occurs within days; however, patients continue to complain of vertigo (26). Dynamic visual acuity (DVA) has been proposed as an outcome measure for testing and rehabilitating the vestibular ocular reflex in acute peripheral hypofunction (27). In the study by Michel et al., the largest improvement in DVA score was found in the early rehabilitation group (*p* < 0.001) rather than in the late rehabilitation group. Both groups showed a statistically significant improvement. However, owing to the lack of randomization, spontaneous remission was not considered.

The DHI questionnaire might introduce a source of bias because translation, interpretation, and cultural adaptation may differ according to country and language. The DHI was translated and cross-culturally adapted to the languages of the RCTs included in the review.

This meta-analysis demonstrated a statistically significant effect of early VRT plus corticosteroids compared with corticosteroids at 1 and 12 months of follow-up.

In this review, VEMP as a parameter of recovery was not a useful outcome measure because only 27/80 patients had abnormal test results at baseline. Ocular VEMPs and cervical VEMPs offer insight into the location of the lesion in pAVS and may provide information on recovery in patients with abnormal test results (28). Other studies found insignificant differences in VEMP between the corticosteroid and vestibular rehabilitation groups (10).

The included studies were characterized by considerable differences in how well the early VRT intervention was described. This is presented in Supplementary Table S2, which was evaluated using the CERT. The scores range from 3 to 17 out of 19 points. In some RCTs, the type of intervention and the specific type of exercise chosen were barely described. The dosage of medication and type and dosage of intervention with early VRT were crucial to the outcome. None of the studies reported compliance or adherence to the interventions, whereas some reported adverse events. The study by Goudakos et al. was the only one to report the occurrence of adverse events.

### 4.1 Clinical safety and compliance

One study reported an adverse event in the form of hyperglycemia, after which corticosteroid treatment was discontinued. None of the included RCTs provided compliance data and only one mentioned compliance.

### 4.2 Limitations of the included RCTs

Meta-analyses of RCTs generally provide reliable results on effective outcome measures and their implications for interventions.

Marioni et al. (18) entitled their publication a “randomized investigation” as they used a computer-generated randomization schedule and as such met the criterion to be considered an RCT. We chose to include their RCT in this systematic review because the data could potentially be used for meta-analysis; however, raw data from their study were not included in the meta-analysis.

Only patients with vestibular neuritis participated in four of the included RCTs. One RCT investigated unilateral peripheral vestibular disorders; however, the diagnostic criteria were set to primarily include vestibular neuritis because normal audiometry was required. Vestibular neuritis often improves spontaneously over time. Because of the self-limiting behavior of this condition, it is challenging to demonstrate a significant effect of early VRT. The solution would be large-scale, well-performed RCTs on vestibular rehabilitation.

### 4.3 Perspectives and future research

Further studies should include a precise description of the applied vestibular rehabilitation program. A consensus on the program is important because it may considerably affect outcomes. As in other medical trials, a lack of research description may disqualify it. Subjective and objective measures of dizziness improvement should include validated questionnaires such as the DHI (at 1 week, 4–6 weeks; and 3, 6, and 12 months). The DHI should be combined with a simple VAS score (0–100) or other simple questions in the acute phase. The objective outcome measures should preferably be video-head impulse testing (v-HIT), caloric lateralization, posturography, or VNG.

## 5 Conclusion

The research questions were then answered; early VRT plus corticosteroids is statistically significant in improving subjective dizziness in pAVS, as illustrated via the meta-analysis of DHI. Early VRT is safe and no serious adverse events have been reported in any RCTs. Caloric lateralization and posturography suggested that early VRT plus corticosteroid was more effective than corticosteroid treatment. However, the studies are inhomogeneous in many respects, which impedes meta-analyses.

## Data availability statement

The original contributions presented in the study are included in the article/Supplementary material, further inquiries can be directed to the corresponding author.

## Author contributions

HAN: Writing – original draft, Writing – review & editing. TG: Writing – review & editing. GB-B: Writing – review & editing. TO: Supervision, Writing – review & editing.
