# Supplementary material for: Early vestibular rehabilitation training of peripheral acute vestibular syndrome—a systematic review and meta-analysis
Source: Front Neurol. 2024 May 30;15:1396891. doi: 10.3389/fneur.2024.1396891 (PMC11169822; doi:10.3389/fneur.2024.1396891)
Supplement: Supplementary file 1 [file Data_Sheet_1.docx]

Supplementary Material

# Supplementary Tables

Supplementary Material: Supplementary Table 1: Search Strategy

| **Resources** | **Search** | **No. Hits** |
| --- | --- | --- |
| Pubmed | ((“vertigo” [MeSH Terms]) OR (“vestibular diseases” [MeSH Terms]) OR (“vestibular neuronitis” [MeSH Terms]) OR (“acute vertigo” [Title/Abstract]) OR (“vestibular neuritis” [Title/Abstract]) OR (“vestibul*” [Title/Abstract]) OR (“acute dizziness” [Title/Abstract])) AND ((“vestibular rehabilitation” [Title/Abstract]) OR (“rehabilit*” [Title/Abstract]) OR (“therap*” [Title/Abstract]) OR (“Therapeutics” [MeSH Terms]) OR (“Rehabilitation” [MeSH Terms])) AND ((“RCT” [Title/Abstract]) OR (“randomized controlled trial” [Title/Abstract]) OR (“randomized controlled trials as topic” [MeSH Terms]) OR (“randomized controlled trials as topic” [MeSH Terms])) | 172 |
| Cinahl | ( MH “Vestibular Diseases+” ) OR ( MH “Neuritis+” ) OR (“acute vertigo” or “vestibular neuritis” or vestibul* or “acute dizziness”) AND ( (MH “Rehabilitation+”) OR (MH “Therapeutics+”) OR (MH “Therapeutic Exercise+”) ) OR (“vestibular rehabilitation” or “vestibular therapy” or rehabilit* or Therap*) AND (MH “Randomized Controlled Trials+” ) OR (“RCT” or “randomized control trial” or “randomized controlled trial”) AND (MH “Vertigo+”) | 269 |
| Embase | 1 exp vertigo/ or acute vertigo.mp. or exp vestibular disorder/ or exp dizziness/  2 exp rehabilitation/  3 randomized controlled trial.mp. or randomized controlled trial/  1, 2, and 3 | 766 |
| Scopus | The reference lists in the five included studies are searched for additional RCTs. | 89 |
| **Total** |  | 1296 |

Supplementary Table 2. Baseline characteristics of included studies

| Study | **Early VRT + steroid** |  |  | **Steroid** |  |  | **Difference between groups** |
| --- | --- | --- | --- | --- | --- | --- | --- |
|  | **Age mean years (SD)** | **No. of patients** | **Gender Male/Female** | **Age mean years(SD)** | **No. of patients** | **Gender Male/Female** |  |
| Goudakos | 53.95  29-79 y | 20 | 13/7 | 51.75  35-79 y | 20 | 11/9 | None |
| Ismail | 49.1 (12.8) | 20 | 10/10 | 47.9 (13.7) | 20 | 12/8 | None |
| Marioni | 45 (7) | 15 | 10/5 | 42 (9) | 15 | 8/7 | None |
| Teggi | 53.5 (9.8) | 20 | 8/12 | 51.4 (9.1) | 20 | 11/9 | None |
| Tokle | 49.5 (14) | 31 | 20/11 | 53.6 (14) | 30 | 23/7 | None |

Supplementary Table 3

Overview of included studies regarding Vestibular Rehabilitation Intervention: Specific programs applied in the included studies. Evaluation by Consensus on Exercise Reporting Template (CERT).

| CERT | Detailed description of: | Goudakos 2014 | Ismail  2018 | Marioni 2013 | Teggi  2009 | Tokle  2020 |
| --- | --- | --- | --- | --- | --- | --- |
| 1 | Exercise equipment | 0* | 0 | 1 | 1 | 1 |
| 2 | Instructor qualifications | 0 | 0 | 1 | 1 | 1 |
| 3 | Individually or group exercise | 1 | 0 | 1 | 1 | 1 |
| 4 | Supervision and delivery | 1 | 0* | 1 | 1 | 1 |
| 5 | How adherence is reported | 1 | 0* | 0* | 0 | 0* |
| 6 | Motivation strategies | 0 | 0 | 0 | 0 | 0 |
| 7a | Decision rules for determining progression | 0 | 0 | 0 | 0 | 1 |
| 7b | How the program was progressed | 0 | 0 | 0 | 0 | 1 |
| 8 | Description of each exercise to enable replication | 1 | 1 | 1 | 0 | 1 |
| 9 | Description of any home program component | 1 | 0 | 1 | 1 | 1 |
| 10 | Any non-exercise components | 1 | 1 | 1 | 1 | 1 |
| 11 | Description of adverse events | 1 | 0* | 1 | 0 | 1 |
| 12 | Setting in which the exercises are performed | 1 | 0 | 1 | 1 | 1 |
| 13 | Description of intervention/program including, number of repetitions, and session duration. | 0* | 0 | 1 | 0* | 1 |
| 14a | Where the exercises generic (one size fits all) or tailored to the individual | 0* | 0* | 1 | 0* | 1 |
| 14b | Where the exercises tailored to the individual | 0* | 0* | 1 | 0* | 1 |
| 15 | Rule for determining the starting level | 0* | 0 | 1 | 0* | 1 |
| 16a | How was adherence or fidelity assessed/measured? | 0* | 0* | 0* | 0 | 1 |
| 16b | Was the intervention delivered as planned? | 1 | 1 | 1 | 0 | 1 |
|  | Total Score | 9 | 3 | 14 | 7 | 17 |

Yes: 1 point; No: 0 points; * Not clearly described.

Supplementary Table 4. Number of patients with abnormal cVEMP test, in the two randomized controlled trials (RCTs).

| Variable | VR^G^ | VR^I^ | Steroid^G^ | Steroid^I^ |
| --- | --- | --- | --- | --- |
| Abnormal cVEMP |  |  |  |  |
| Baseline | 7/20 | 8/20 | 4/20 | 8/20 |
| 1 month | 5/20 | 6/20 | 4/20 | 6/20 |
| 6 months | 0/20 | 2/20 | 0/20 | 0/20 |
| 12 months | 0/20 | 0/20 | 0/20 | 0/20 |

^G^: Goudakos, ^I^: Ismail
